# 贝林妥欧单抗治疗复发难治急性淋巴细胞白血病13例疗效及不良反应分析

**DOI:** 10.3760/cma.j.issn.0253-2727.2023.06.015

**Published:** 2023-06

**Authors:** 亚军 石, 英 汉, 莹 王, 东锋 毛, 君玲 张, 瑞 葸, 海 白, 涛 吴

**Affiliations:** 解放军联勤保障部队第九四〇医院血液科，兰州 730050 Department of Hematology, the 940th Hospital of Joint Logistics Support Force of Chinese People's Liberation Amy, Lanzhou 730050, China

复发难治急性B淋巴细胞白血病（R/R B-ALL）的治疗一直是国内外血液学领域研究的热点，近年新技术及新药不断涌现，如CAR-T细胞、贝林妥欧单抗及靶向CD22抗体偶联药物奥加伊妥珠单抗等[1]–[3]。其中，贝林妥欧单抗在国内上市不久，使用相对较少，国内仅见个案报道。因此，我们总结我院贝林妥欧单抗的使用情况，旨在分析该药的有效率、不良反应及注意事项，为临床医师提供参考。

## 病例与方法

1. 病例资料：回顾性收集解放军联勤保障部队第九四〇医院血液科于2021年6月至2022年8月使用贝林妥欧单抗治疗的13例复发难治B-ALL患者的临床资料（共计使用14例次单抗），分析其治疗的效果及并发症。

2. 观察指标：一般基线资料包括患者的年龄、性别、病程、贝林妥欧单抗使用前缓解情况；疗效指标包括使用贝林妥欧单抗前后血常规、肝肾功能、骨髓细胞形态学、微小残留病（MRD）变化及BCR-ABL融合基因水平等。

3. 贝林妥欧单抗具体用药剂量及周期：体重≥45 kg的患者第1～7天 9 µg/d，第8～14天或第8～28天 28 µg/d。体重<45 kg的患者，第1～7天5 µg·m^−2^·d^−1^，第8～14天或第8～28天 15 µg·m^−2^·d^−1^。第1天及第8天用药前1 h均给予静脉滴注地塞米松，成人20 mg，儿童减量为10 mg。

4. B-ALL缓解的定义：骨髓细胞形态学原始细胞小于5％，外周血中无原始细胞[4]。

5. B-ALL难治、复发的定义：参照《中国成人急性淋巴细胞白血病诊断与治疗指南（2021年版）》和相关指南[4]–[5]，骨髓复发指ALL完全缓解（CR）后外周血或骨髓中再次出现原始细胞（比例大于5％）或出现髓外病变。难治指诱导治疗结束未能取得CR或CR伴血细胞不完全恢复（CRi）；第1次CR后6个月内复发；第2次CR后6个月以上复发且经诱导治疗未能缓解；2次及以上复发；出现髓外病变。MRD复发指MRD阴性后流式细胞术再次检测到大于10^−4^（0.01％）水平的肿瘤细胞（骨髓达CR）或融合基因转为阳性。分子学复发指融合基因转为阳性。

6. 不良反应评价：贝林妥欧单抗不良反应中血液学毒性及非血液学毒性依据CTCAE 5.0标准，细胞因子释放综合征（CRS）分级依据Lee分级标准[6]。

7. 统计学处理：计数资料以例数及百分比表示。

## 结果

一、患者一般临床资料

共13例R/R B-ALL患者使用贝林妥欧单抗，共使用14例次。其中男11例，女2例。中位年龄17（7～17）岁；复发14例次，包括骨髓复发8例次（57.1％），MRD复发（包括分子学复发2例次）6例次（42.9％）；难治7例次（7/14，占50.0％）（表1）。

二、疗效情况

13例R/R B-ALL患者中，例1在3个周期贝林妥欧单抗使用过程中，肝肾功能均正常，因经济情况，每次使用14 d，于2022年2月28日复查血常规大致正常，骨髓细胞形态学达CR，MRD阴性，第2和第3个周期为巩固治疗，因此计为1次。例2在移植前后共计使用2个周期贝林妥欧单抗，第1个周期是因骨髓复发使用，第2个周期因移植后MRD阳性使用，因此单抗使用计为两次。总共使用14例次贝林妥欧单抗（表1）。共计3例次患者使用28 d，1例次使用20 d（治疗无效提前结束），9例次因经济原因只使用14 d，1例次使用10 d（提前结束）。

骨髓缓解情况：使用单抗前，共8例患者骨髓复发，7例患者难治，使用单抗后，6例患者骨髓达CR，其中，4例达MRD阴性，2例MRD阳性；2例患者未缓解。MRD转阴情况：使用单抗前，共6例患者仅MRD阳性（骨髓已达CR，包括2例融合基因阳性），使用后5例患者MRD阴性，1例出现骨髓复发，疾病进展。BCR-ABL P210定量水平：使用单抗前，共2例患者仅BCR-ABL基因定量阳性（流式细胞术MRD阴性），使用后1例患者基因定量为0，另1例患者基因定量达到MR4.5。每例患者使用贝林妥欧单抗的疗效见表1。

**表1:** 13例急性B淋巴细胞白血病（B-ALL）患者的临床特征及疗效

例号	性别	年龄（岁）	融合基因	病程（月）	移植与否	单抗治疗前复发情况	单抗治疗前难治情况	单抗使用时间	使用前BM及MRD情况	使用后BM情况	使用后MRD及基因定量情况
1	男	10	IKZF3-8（IK6）MYOM2和TET2点突变	18	移植后11个月余	骨髓及睾丸复发	难治	14 d	①BM：5.6% MRD：2.2% ②BM：0 MRD：0 ③BM：0 MRD：0	① 0 ② 0 ③ 0	①MRD：0 ②MRD：0 ③MRD：0
2	男	12	TCF3-HLF	11	①移植前 ②移植后 4个月余	①骨髓复发 ②MRD复发	难治	①28 d ②14 d	①BM：29.5% MRD：34.2% ②BM：0 MRD：0.64%	① 0 ② 0	①MRD：0 ②MRD：0
3	男	68	BCR-ABL P210/P230	36	否	骨髓复发	否	14 d	BM：92.8% MRD：89.7%	0	MRD：18.4%
4	男	52	BCR-ABL P190	18	否	骨髓复发	否	14 d	BM：80.8% MRD：36.0%	0	MRD：0
5	男	15	BCR-ABL P210	8	是	分子学复发（MRD复发）	否	14 d	BM：0 MRD：0 P210：0.0346	0	MRD：0 P210：0
6	女	26	无	4	是	骨髓复发	难治	28 d	BM：56.0% MRD：36.2%	0	MRD：1.17%
7	男	49	BCR-ABL P210	9	是	分子学复发（MRD复发）	否	14 d	BM：0 MRD：0 P210：0.0124	0	MRD：0 P210：0.000268
8	男	70	无	10	否	MRD复发	否	14 d	BM：0 MRD：0.03%	0	MRD：0
9	男	12	TCF3-PBX1	8	否	MRD复发	否	20 d	BM：0 MRD：7.6%	55%	MRD：5.91%
10	男	7	无	54	否	骨髓复发	难治	28 d	BM：10% MRD：9.8%	0	MRD：0
11	男	17	WT1	29	否	骨髓复发	难治	14 d	BM：85.2% MRD：56.2%	80%	MRD：55.6%
12	女	48	BCR-ABL P190复杂核型	12	否	骨髓复发	难治	10 d	PB：58% BM：44% MRD：63%	40% PB：24%	MRD：未查
13	男	7.5	无	4	否	MRD复发	难治	14d	BM：0 MRD：0.25%	0	MRD：0

注 MRD：微小残留病；BM：骨髓；PB：外周血。BM情况数据为原始细胞比例

三、不良反应

1. 非血液学不良反应：11例次出现输注相关的发热；1例出现癫痫；1例出现三叉神经痛；1例出现头痛；3例出现细胞因子释放综合征（CRS），其中1级1例，2级2例；2例出现皮疹；4例出现转氨酶升高，其中1级转氨酶升高2例，2、3级各1例；1例出现1级胆红素升高；1例患者单抗治疗结束12 d后出现卡氏肺囊虫肺炎（PCP）。

2. 血液学不良反应：8例次发生Ⅲ～Ⅳ级中性粒细胞减少，6例次发生Ⅲ～Ⅳ级血红蛋白减少，5例次发生Ⅲ～Ⅳ级血小板减少。发生Ⅳ级中性粒细胞减少、血红蛋白及血小板减少患者使用单抗后骨髓均未缓解，出现疾病迅速进展。

## 讨论

贝林妥欧单抗可与B细胞表面的CD19和T细胞表面的CD3结合，激活人体内源性T细胞，引起CD19阳性的原始细胞裂解[7]–[8]，多项研究显示其在治疗R/R B-ALL中的有效性[9]。

Couturier等[9]分析了26例R/R Ph^+^ B-ALL患者，使用贝林妥欧单抗联合普纳替尼后96.15％的患者达CR，88.46％的患者达MRD阴性。TOWER研究亦证实贝林妥欧单抗的缓解率及生存率显著高于化疗[10]。Locatelli等[11]在首次复发B-ALL患儿中，发现贝林妥欧单抗可使93％的患儿MRD转阴性。国内Ⅲ期临床研究证实，贝林妥欧单抗治疗R/R Ph^−^ B-ALL患者，CR/部分血液学恢复的CR（CRh）率45.6％，MRD缓解率83％[12]。结合本中心情况，骨髓复发患者中，单抗治疗后75％达骨髓CR，50％达MRD阴性；MRD阳性患者中，使用单抗后83.3％患者MRD达阴性。从整体情况看，贝林妥欧单抗治疗R/R B-ALL有较高的缓解率。

本中心25％骨髓复发的患者贝林妥欧单抗无效，1例MRD阳性患者单抗治疗期间出现骨髓复发，疾病进展。Zhao等[13]采用贝林妥欧单抗治疗44例R/R B-ALL患者，无效占45％，研究认为贝林妥欧无效可能是CD19表达缺失引起，包括CD19突变、CD19突变等位基因特异性表达、CD19 RNA低表达等[13]–[14]。因此，贝林妥欧单抗治疗期间，建议每周复查外周血细胞形态，每两周复查骨髓形态或MRD，尽早发现对单抗耐药的患者，及时停药，减轻患者经济负担。

贝林妥欧单抗的血液学不良反应方面，本中心57.1％的患者发生Ⅲ～Ⅳ级中性粒细胞减少，这与国内Ⅲ期研究数据大致相同（58.9％）[12]，但Ⅲ～Ⅳ级血红蛋白减少（42.9％）和血小板减少（35.7％）发生率较TOWER研究高[10]，且主要发生在单抗使用后骨髓仍未缓解的患者，考虑与疾病进展、高肿瘤负荷抑制骨髓造血相关。非血液学不良反应方面，本中心78.6％的患者出现发热，28.6％患者出现转氨酶升高，分别高于TOWER研究的55％和15％[10]，可能与样本较少有关。14.3％的患者出现皮疹，这与TOWER研究的12％接近。与贝林妥欧单抗相关的头痛、癫痫和三叉神经痛等神经系统毒性发生率为21.4％，这与Couturier等[9]报道的23.08％及TOWER研究[10]报道的23％相一致。本中心21.4％的患者出现CRS，且主要发生在肿瘤负荷高的患者，这与RIALTO研究的20％相一致[15]，但高于Couturier等[9]报道的11.53％及TOWER研究的14％[10]。不良反应中，尤其要注意CRS发生在高肿瘤负荷的患者，对原始细胞大于20％的患者，建议使用单抗第1周内给予地塞米松、水化、碱化、利尿等减轻CRS症状，对于持续高热、伴呼吸衰竭等严重症状的患者建议早期给予托珠单抗减轻炎症因子释放，必要时给予呼吸机辅助呼吸[16]–[17]。

需要警惕单抗相关的中枢神经系统和肺部感染的严重不良反应，本中心1例患儿在第2次单抗治疗期间出现癫痫发作，停治疗后癫痫未再发作，考虑可能与爬坡时间不够相关，建议贝林妥欧单抗从小剂量开始爬坡治疗，1周后再增加单抗剂量。1例患儿单抗治疗结束12 d后出现肺部重度感染，合并Ⅰ型呼吸衰竭，影像学考虑PCP，这可能与贝林妥欧单抗抑制体液免疫，增加感染风险相关。国内亦报道1例64岁男性经TKI联合贝林妥欧单抗治疗后出现PCP[18]。因此，建议在单抗治疗结束，对异基因造血干细胞移植（allo-HSCT）后、高龄、合并基础病等高危人群予以复方新诺明预防PCP。同时需要关注的是，本中心有两例TCF3阳性B-ALL患儿，其中一例TCF3-HLF阳性，骨髓复发使用单抗后桥接allo-HSCT，但移植后出现MRD阳性，再次给予单抗后转阴。TCF3-HLF是由t（17；19）（q22；p13）产生，多见于儿童，化疗敏感度低，复发率高，预后差，长期生存率不到10％[19]–[20]。另一例TCF3-PBX1阳性，化疗期间出现复发，使用单抗无效，结局差。TCF3-PBX1是由t（1；19）产生，国内学者报道了137例伴TCF3-PBX1阳性的B-ALL，发现该基因多见于青少年及年轻成人患者，5年总体生存率68.6％，CR_1_后行allo-HSCT能显著提高患者5年生存率，其中年龄、初诊染色体伴有t（1；19）（q23；p13）和诱导缓解后MRD阳性是影响预后的独立危险因素[21]。因此，建议对TCF3-HLF或TCF3-PBX1阳性的B-ALL患者达CR后尽早行allo-HSCT，移植后密切关注MRD，必要时予以贝林妥欧单抗维持治疗降低复发风险。

总之，贝林妥欧单抗治疗复发难治B-ALL的缓解率较高，不良反应较小，可优先推荐用于预计桥接移植的MRD阳性患者。
